# Effects of navigated TMS on object and action naming

**DOI:** 10.3389/fnhum.2014.00660

**Published:** 2014-09-02

**Authors:** Julio C. Hernandez-Pavon, Niko Mäkelä, Henri Lehtinen, Pantelis Lioumis, Jyrki P. Mäkelä

**Affiliations:** ^1^Department of Biomedical Engineering and Computational Science, Aalto University School of ScienceEspoo, Finland; ^2^BioMag Laboratory, HUS Medical Imaging Center, Helsinki University Central HospitalHelsinki, Finland; ^3^Epilepsy Unit, Department of Pediatric Neurology, Helsinki University Central HospitalHelsinki, Finland

**Keywords:** transcranial magnetic stimulation, speech mapping, left hemisphere, object naming, action naming

## Abstract

Transcranial magnetic stimulation (TMS) has been used to induce speech disturbances and to affect speech performance during different naming tasks. Lately, repetitive navigated TMS (nTMS) has been used for non-invasive mapping of cortical speech-related areas. Different naming tasks may give different information that can be useful for presurgical evaluation. We studied the sensitivity of object and action naming tasks to nTMS and compared the distributions of cortical sites where nTMS produced naming errors. Eight healthy subjects named pictures of objects and actions during repetitive nTMS delivered to semi-random left-hemispheric sites. Subject-validated image stacks were obtained in the baseline naming of all pictures before nTMS. Thereafter, nTMS pulse trains were delivered while the subjects were naming the images of objects or actions. The sessions were video-recorded for offline analysis. Naming during nTMS was compared with the baseline performance. The nTMS-induced naming errors were categorized by error type and location. nTMS produced no-response errors, phonological paraphasias, and semantic paraphasias. In seven out of eight subjects, nTMS produced more errors during object than action naming. Both intrasubject and intersubject analysis showed that object naming was significantly more sensitive to nTMS. When the number of errors was compared according to a given area, nTMS to postcentral gyrus induced more errors during object than action naming. Object naming is apparently more easily disrupted by TMS than action naming. Different stimulus types can be useful for locating different aspects of speech functions. This provides new possibilities in both basic and clinical research of cortical speech representations.

## Introduction

Transcranial magnetic stimulation (TMS) is a noninvasive technique where a strong and brief magnetic pulse is delivered to the brain and induces electrical currents. This produces depolarization of cellular membranes and neuronal activation (Barker et al., 1985; Ilmoniemi et al., 1999). TMS has become an important tool for studying speech and language at both the cognitive and neural level (Devlin and Watkins, 2007). TMS may produce both inhibition and facilitation during different phases of speech processing either by directly stimulating a specific speech-related cortical region or indirectly through intracortical networks (Epstein, 1998). TMS has been used for studying the functional localization of speech in healthy subjects, with variable results (Pascual-Leone et al., 1991; Epstein et al., 1999; Devlin and Watkins, 2007; Vigliocco et al., 2011).

Navigated TMS (nTMS) is considered the state-of-the-art technique in performing TMS studies (Siebner et al., 2009). In nTMS, the stimulated cortical site can be defined anatomically from the individual's brain magnetic resonance images (MRI). In addition, orientation and strength of the induced electric field can be estimated (Siebner et al., 2009; Ruohonen and Karhu, 2010). The information provided by nTMS is useful for surgical planning, and it can be transferred into the operating theater via surgical neuronavigation systems.

So far, nTMS has been used in preoperative localization of the motor cortex (Picht et al., 2009; Vitikainen et al., 2009). It localizes the cortical representations of hand muscles as accurately as direct cortical stimulation (DCS) (Picht et al., 2011; Krieg et al., 2012) and more accurately than functional magnetic resonance imaging (fMRI) (Forster et al., 2011; Krieg et al., 2012). In addition, neuromodulation of Broca's area in speech-related tasks is reported to be more robust by nTMS than by conventional TMS based on external landmarks on the head (Kim et al., 2013). These results motivated us to develop a protocol for preoperative localization of speech-related brain areas by utilizing object naming and nTMS (Lioumis et al., 2012). This novel approach has been compared to DCS during awake craniotomy (Picht et al., 2013). The results imply that nTMS is remarkably sensitive but relatively non-specific in detecting the sites producing speech disturbance in DCS. Discordance between nTMS and DCS was observed particularly in the posterior cortical regions (Picht et al., 2013; Tarapore et al., 2013). Preoperative speech mapping by nTMS can give important a priori information to the neurosurgeons. It may aid in objective preoperative risk-benefit balancing of the planned surgery, more targeted and smaller craniotomies, faster and safer intraoperative mapping, and safer surgeries for patients that cannot undergo awake craniotomy (Picht et al., 2013). Recently, two studies have used object naming and nTMS to compare language mapping on patients with brain tumors and healthy subjects, suggesting tumor-induced plasticity of speech representation areas (Krieg et al., 2013; Rösler et al., 2013). Thus, better understanding of the effects of TMS during naming tasks may have an impact on surgery planning and provide information about the cortical organization of speech in general.

Picture naming has been extensively studied in both healthy subjects and patients with various neurological diseases. In magnetoencephalography (MEG) and fMRI studies on healthy subjects, action and object naming activate cortical networks including left inferior frontal gyrus, left dorsal premotor, bilateral occipitotemporal, and bilateral parietal areas (Sörös et al., 2003; Petrovich Brennan et al., 2007; Liljeström et al., 2008, 2009). Some functional neuroimaging studies suggest different cortical representations of action and object naming (for a review, see Vigliocco et al., 2011). It has been suggested that action naming activates particularly the left premotor (Valyear et al., 2007; Canessa et al., 2008), parietal (Noppeney et al., 2005), and frontal cortex (Vigliocco et al., 2011), whereas object naming activates the left temporal areas most strongly (Vigliocco et al., 2011). In line, patients with aphasia due to lesions in left frontal areas have shown more severe deficits in action naming, whereas lesions in the left temporal areas are associated with deficits in object naming (Mätzig et al., 2009; Vigliocco et al., 2011).

Action naming appears to be a demanding process that requires more extensive neural processing than object naming (Mätzig et al., 2009). TMS studies indicate that left prefrontal and motor cortices are involved in processing verbs and actions (Pulvermüller et al., 2005; Cappelletti et al., 2008; Gerfo et al., 2008). However, the areas stimulated in (Cappelletti et al., 2008) and (Gerfo et al., 2008) do not match with areas of greater activation for verbs and nouns in imaging studies using similar tasks (Vigliocco et al., 2011). Intraoperative cortical mapping by DCS during awake craniotomy in tumor surgery has systematically revealed widely distributed and highly individual effective cortical sites (Whitaker and Ojemann, 1977; Sanai et al., 2008; Corina et al., 2010) and dissociation of sites inducing errors in action and object naming (Corina et al., 2005; Lubrano et al., 2014). The cortical sites activated specifically by action naming resided mainly in the parietal cortex (Corina et al., 2005).

We mapped the left-hemispheric speech-related areas by nTMS during object and action naming tasks. The induced errors during object and action naming were categorized by type and location of the stimulated cortical site, and compared with each other. We were particularly interested to see if action naming would be interfered more by nTMS in the posterior cortical areas, where discordant results between nTMS and DCS were seen in an object naming task (Picht et al., 2013; Tarapore et al., 2013). If so, action naming tasks might add information in detecting speech-related cortical areas from this region by means of nTMS, as suggested in previous studies (Corina et al., 2005; Noppeney et al., 2005). As action naming is considered more demanding than object naming (Berndt et al., 1997; Mätzig et al., 2009), we hypothesized that action naming would be more easily disturbed by nTMS than object naming.

## Methods

### Subjects

Eight neurologically normal right-handed subjects (native speakers of Finnish; mean age 26 ± 2 years, four females) participated in the study. The subjects had normal or corrected-to-normal vision. The study was approved by the Ethics Committee of Helsinki University Central Hospital and was in compliance with the declaration of Helsinki. The subjects gave their written informed consent before the experiments.

### Object and action naming

**Figure 1:**
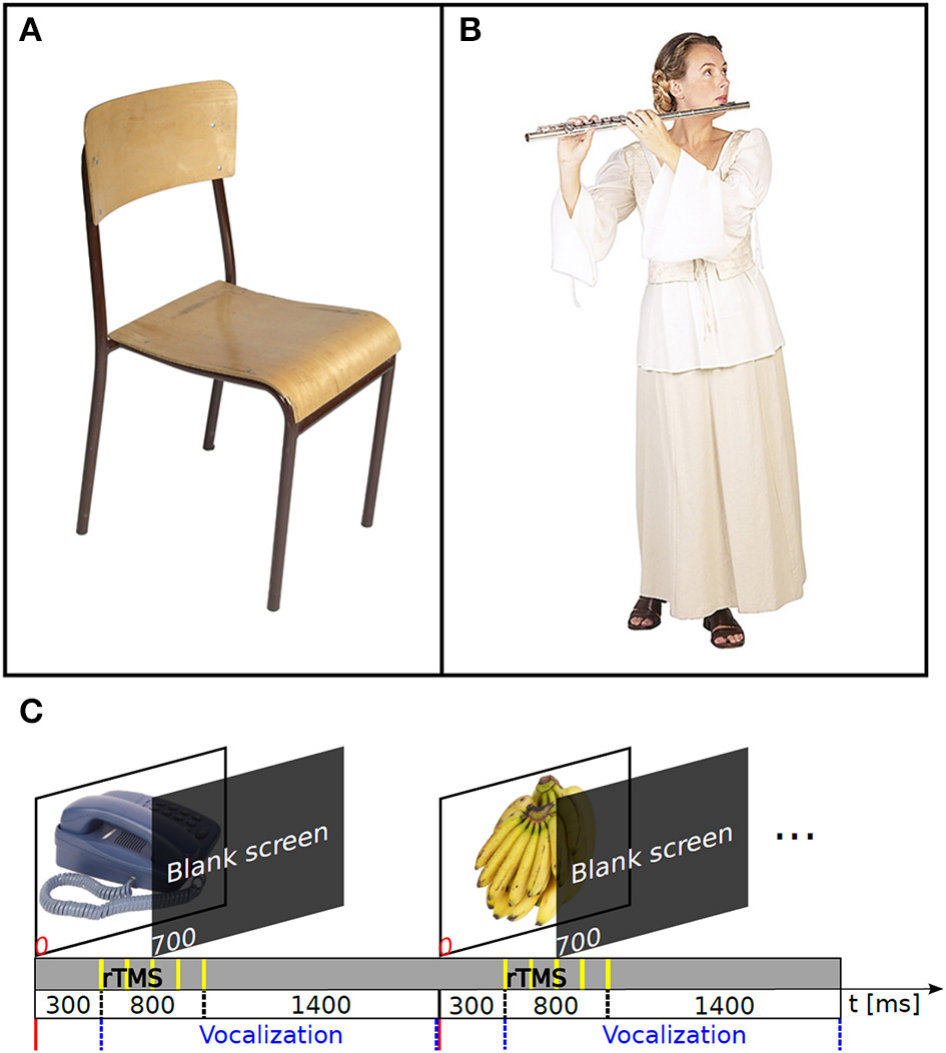
**The nTMS speech mapping method**. An example of images used in object **(A)** and action naming **(B)**. **(C)** Timeline of the events in the nTMS speech mapping. The interpicture interval was 2500 ms.

We used two sets of color pictures with a white background, one with 131 images depicting objects and another with 98 images depicting actions. Object images illustrated a simple object (e.g., a chair; Figure 1A; see also the video in the Supplementary Material). The action images represented a simple event (e.g., playing an instrument; Figure 1B). The subjects were asked to name objects or actions in Finnish as quickly and precisely as possible. Two subjects performed action naming before object naming. The experiment consisted of two baseline sessions without nTMS (one for object and another for action naming) and two nTMS sessions (one with object naming and another with action naming). All sessions were video-recorded for offline analysis. The baseline sessions were done before the nTMS sessions. Images that were unfamiliar or named incorrectly in the baseline session were removed from the image set used during nTMS (see the Supplementary Table). Thus, only fluently named images were used during the nTMS sessions. The numbers of rejected object and action images did not differ significantly (Mann–Whitney *U*-test; *p* = 0.26). The images were displayed in random order within the object naming and action naming sessions (see the Supplementary Video). For each subject, all TMS measurements were performed in a row.

### Stimulation

Two recording setups were used. In setup 1, we used eXimia Navigated Brain Stimulation (NBS) version 3.2 (Nexstim Ltd., Helsinki Finland); for details, see Lioumis et al. (2012). In setup 2, we used eXimia NBS version 4.3 and a commercial speech-mapping module (NexSpeech, Nexstim Ltd., Helsinki Finland). Both navigation systems calculate the strength of the maximum electric field that is overlaid on-line on the 3-D reconstruction of the individual's brain (Ruohonen and Karhu, 2010). Each stimulation site is tagged to the MR image for subsequent studies.

All stimulations were done with a biphasic figure-of-eight coil. The outer diameter of the coil was 70 mm. The resting motor threshold (MT) was determined from the right abductor pollicis brevis (APB) muscle, and the strength of the induced electric field at the cortex was registered. These electric fields varied between 40 and 100 V/m at approximately 25 mm from the head surface (i.e., at the navigation depth). The stimulus intensity for the speech mapping was adjusted to produce roughly as strong electric field to perisylvian cortical regions. Navigated TMS of temporal areas occasionally produces some discomfort. However, we were particularly meticulous to avoid such unpleasantness; if the stimulation caused discomfort to the subject due to muscle contraction (in a short test session before the actual measurements), the stimulation intensity was lowered in decrements of 5–10% until it was tolerable. Moreover, the experimental setup has been validated by DCS, where discomfort due to scalp or muscle stimulation is not an issue, and found to match well with DCS particularly in ventral anterior areas (see e.g., Picht et al., 2013; Tarapore et al., 2013; Krieg et al., 2014). Consequently, the stimulation intensity varied somewhat across subjects (80–110% of the APB MT; 30–40% of the stimulator output). The stimulation was done with nTMS trains of five pulses at 5 Hz (Epstein et al., 1996; Lioumis et al., 2012). The subjects wore earplugs during all sessions.

The object and action pictures were displayed for 700 ms on a computer screen once every 2.5 s. The nTMS trains were delivered with a 300 ms delay after the picture onset (Figure 1C; Supplementary Video). The nTMS onset time was chosen on the basis of MEG studies on dynamics of cortical language processing (Salmelin et al., 2000; Sörös et al., 2003); essentially we did not want to interfere with the visual inspection, but to disturb other stages of language production (e.g., conceptual processing, lexical selection, phonological encoding, and articulatory preparation). The coil was hand-held and it was moved freely between the pulse trains. Approximately 200 sites were stimulated in the left hemisphere by moving the coil semi-randomly in between the trains of pulses, following a grid-like pattern so that the tested target sites covered systematically a wide fronto-temporo-parietal cortical area. The same areas were stimulated for both tasks. The orientation of the coil was adjusted to induce current primarily perpendicular to the fibers of the temporalis muscle to minimize muscle twitching, and secondarily perpendicular to the sulcus at the stimulation target. The cortical sites where nTMS-induced errors were observed online and were revisited to evaluate the repeatability of the effect (see Supplementary Video). On average, 257 stimulus trains were delivered to the left hemisphere during object naming and 243 during action naming in each subject. The maximum difference between repetitions for two different images was one, as the content of the subject-validated image stack was randomized each time a new round of the images started.

### Data analysis

A neuropsychologist with expertise in effects of DCS on speech (HL) analyzed naming performance in the recorded videos. During the analysis, the stimulation sites were not visible. The baseline naming responses were compared with those recorded during nTMS. The observed errors were categorized as no-response errors, semantic paraphasias, and phonological paraphasias according to previous studies (Corina et al., 2010; Picht et al., 2013; Rösler et al., 2013). *No-response errors*: stimulation leads to a complete lack of naming response. *Phonologic paraphasias*: characterized by unintended phonemic modification of the target word. The spoken word resembles the target word, but is phonetically different. For example the target word “pants” is replaced with “plants.” *Semantic paraphasias*: errors in which the patient substitutes a semantically related or associated word for the target word. For example, the target word “cow” is replaced by the word “horse.” When a naming error occurred, the corresponding nTMS location was marked as speech-related and tagged by the observed error type. Thereafter, the nTMS sites eliciting naming errors were grouped into cortical regions. For the anatomical labeling, we used the anatomical atlas shown in Corina et al. (2010) as in previous publications (Lioumis et al., 2012; Picht et al., 2013). The cortical surface of each subject was separated into anatomical regions according to this template.

The statistical significance of the results were evaluated both in single-subject and group level. For the single-subject analysis, the statistical significance of the observed effects of nTMS on performance in the naming tasks was evaluated separately for each subject and stimulated area. The null hypothesis was that the observed errors occur due to chance. If so, the number of observed errors should follow a Poisson distribution with the parameter λ = number of observed errors (per area)/the total number of nTMS trials (per area). The probability that the observed number of naming errors in an area could have arisen by chance rather than due to the effect of nTMS was computed by comparing the number of observed errors with one million simulated Poisson samples. The number of samples in the simulated data that were greater than or equal to the observed number of errors gives the probability of the case that the observed effect could have occurred by chance. The significance level was set at 5%. False discovery rate (FDR) was applied on the *p*-values collected from the area wise analyses of each subject to correct for multiple comparisons (Storey, 2002).

For the group level analysis, the 2-tailed Mann–Whitney *U*-test was used to compare the number of errors during object and action naming. The statistical analysis was done to the total number of naming errors in the left hemisphere, and within each error type and gyrus. The significance level was set at 5%.

**Figure 2:**
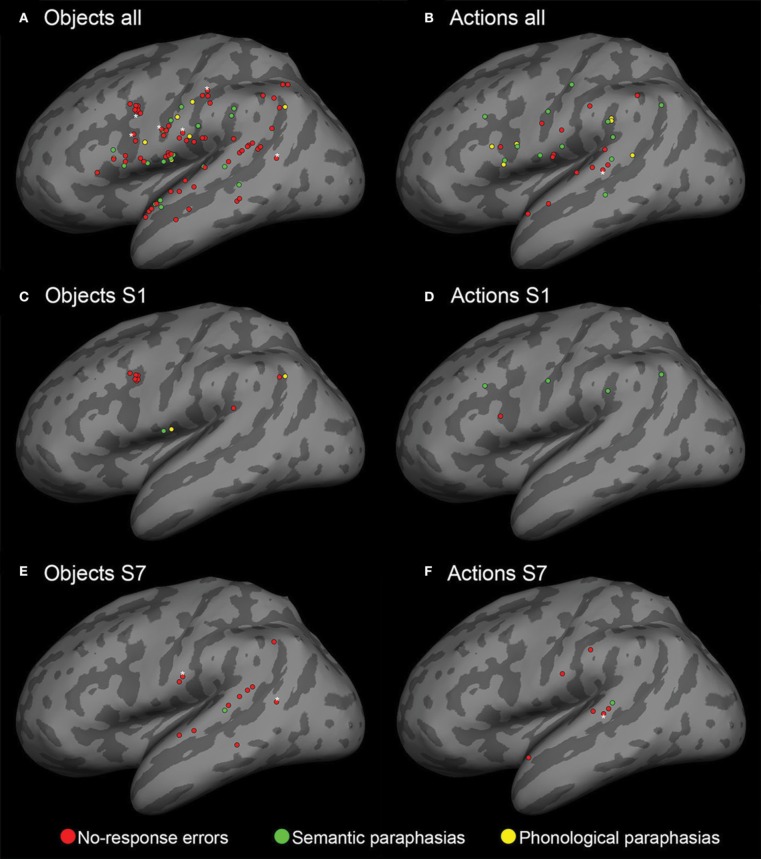
**Cortical sites for both object and action naming errors visualized on an inflated reconstruction of the cortex**. Red spheres: no-response errors; green spheres: semantic paraphasias; yellow spheres: phonological paraphasias. (**A,B**) All cortical sites that elicited nTMS-induced naming errors in the subjects. **(C–F)** Individual data from subjects S1 and S7. The number, type, and location of the naming errors vary between subjects. The white asterisks indicate the sites of repeated errors at the same location.

To visually summarize the speech mapping results, the stimulation sites that were associated with naming errors from all eight subjects were projected on the standardized MNI brain template (Mazziotta et al., 2001), using FSL (Smith et al., 2004; Woolrich et al., 2009; Jenkinson et al., 2012) and FreeSurfer (Fischl et al., 1999) softwares. The brain was segmented from the individual T1-weighted MRIs of each subject and registered with the standard brain template in MNI space. Thereafter, the coordinates of the naming error locations were projected into the MNI space, using the transformation matrix given by the registration and overlaid with the inflated cortical surface of the MNI brain template (Figure 2).

## Results

**Figure 3:**
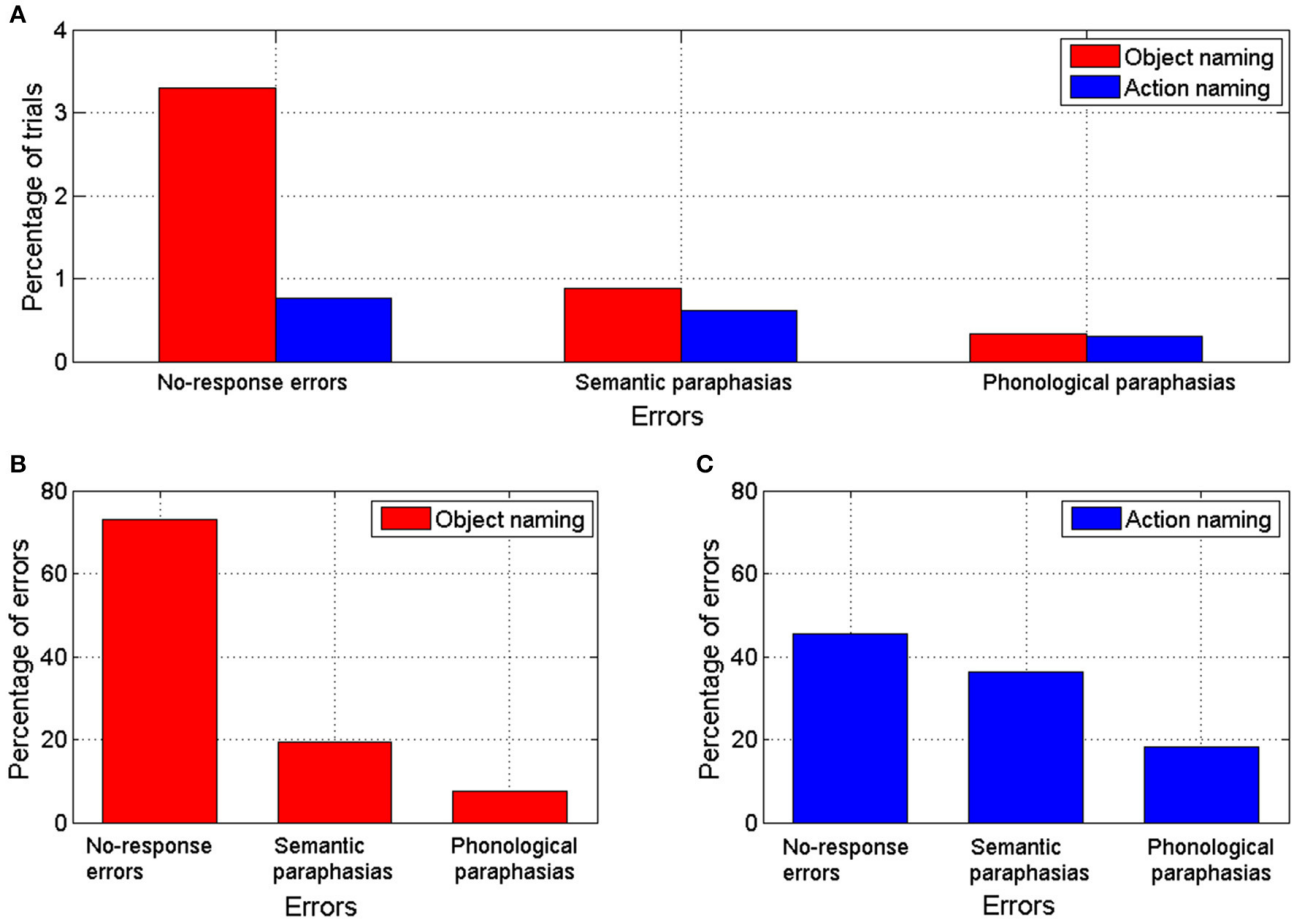
**(A)** Percentage of trials with errors out of the total number of trials during nTMS for object and action naming tasks. **(B)** Percentage of errors of each error category out of the total number of naming errors produced by nTMS during object naming task. **(C)** Percentage of errors of each error category out of the total number of naming errors produced by nTMS during action naming task.

Overall 93 nTMS trains (4.5% from a total of 2056 trains) induced errors during object naming. During action naming, 33 nTMS trains (1.7% from a total of 1944 trains) induced errors (Figure 3A). In seven out of eight subjects, TMS elicited more object naming than action naming errors. In one subject, the total number of induced errors was equal in both (Table 1). Naming errors were induced when nTMS was delivered to angular gyrus (anG), inferior frontal gyrus (IFG), middle frontal gyrus (MFG), postcentral gyrus (PoG), precentral gyrus (PrG), superior temporal gyrus (STG), middle temporal gyrus (MTG), and supramarginal gyrus (SMG) (Table 1 and Figure 2).

**Table 1:** **Observed nTMS-induced errors for all subjects during object and action naming in different cortical areas (object; action)**.

**Areas/Subjects**	**No-response errors**								**Semantic paraphasias**								**Phonological paraphasias**								**Total errors**
	**S1**	**S2**	**S3**	**S4**	**S5**	**S6**	**S7**	**S8**	**S1**	**S2**	**S3**	**S4**	**S5**	**S6**	**S7**	**S8**	**S1**	**S2**	**S3**	**S4**	**S5**	**S6**	**S7**	**S8**	
anG	1; 0	1; 0	0; 0	0; 0	0; 0	0; 0	0; 0	0; 0	0; 1	0; 0	0; 0	0; 0	0; 0	0; 0	0; 0	0; 0	0; 0	0; 0	0; 0	0; 0	0; 0	0; 0	0; 0	0; 0	2; 1
IFG	2; 1	3; 0	3; 0	0; 0	0; 0	1; 0	0; 1	7; 0	0; 1	0; 0	0; 0	0; 0	0; 2	0; 0	0; 0	1; 0	0; 0	0; 0	0; 0	0; 0	0; 1	1; 1	0; 0	0; 0	18; 7
MFG	2; 0	1; 0	0; 0	0; 0	0; 0	0; 0	0; 0	0; 0	0; 0	0; 0	0; 0	0; 0	0; 0	0; 0	0; 0	0; 0	0; 0	0; 0	0; 0	0; 0	0; 0	0; 1	0; 0	0; 0	3; 1
PoG	0; 0	2; 0	0; 0	0; 0	2; 0	2; 0	3; 2	5; 1	1; 0	1; 0	0; 0	1; 0	0; 0	1; 0	0; 0	1; 0	1; 0	0; 1	0; 0	0; 0	0; 0	3; 0	0; 0	0; 0	23; 4
PrG	2; 0	1; 0	4; 1	0; 0	0; 0	0; 0	0; 0	1; 0	0; 1	1; 1	0; 0	0; 1	0; 0	1; 0	0; 0	0; 0	0; 0	0; 0	0; 0	0; 0	0; 0	1; 0	0; 0	0; 0	11; 4
STG	0; 0	0; 0	2; 3	6; 0	1; 1	0; 0	5; 4	2; 0	0; 0	0; 0	3; 1	2; 0	0; 0	0; 0	0; 1	0; 0	0; 0	0; 1	0; 0	0; 0	0; 0	0; 0	0; 0	0; 0	21; 11
MTG	0; 0	0; 0	1; 0	0; 0	0; 0	0; 0	5; 0	0; 0	0; 0	0; 0	1; 1	0; 0	0; 0	0; 0	0; 0	0; 0	0; 0	0; 0	0; 0	0; 0	0; 0	0; 0	0; 0	0; 0	7; 1
SMG	1; 0	1; 0	0; 1	0; 0	0; 0	0; 0	0; 0	1; 0	0; 1	1; 1	0; 0	0; 0	1; 0	1; 0	1; 0	0; 0	1; 0	0; 1	0; 0	0; 0	0; 0	0; 0	0; 0	0; 0	8; 4
Total errors (object;action)	8; 1	9; 0	10; 5	6; 0	3; 1	3; 0	13; 7	16; 1	1; 4	3; 2	4; 2	3; 1	1; 2	3; 0	1; 1	2; 0	2; 0	0; 3	0; 0	0; 0	0; 1	5; 2	0; 0	0; 0	
	68; 15								18; 12								7; 6								93; 33

Notation: anG, angular gyrus; IFG, inferior frontal gyrus; MFG, middle frontal gyrus; PoG, postcentral gyrus; PrG, precentral gyrus; STG, superior temporal gyrus; MTG, middle temporal gyrus; SMG supramarginal gyrus.

In the object naming task, 25% of the sites associated with naming errors were located in the PoG, 23% in the STG, 19% in the IFG, 12% in the PrG, 9% in the SMG, 8% in the MTG, 3% in the MFG, and 2% in the anG (Table 1 and Figure 2). A subanalysis by type showed that 24% of the no-response errors (73% of all naming errors, see Figure 3B) were induced from the IFG, 24% from the STG, 21% from the PoG, and 12% from the PrG (Table 1). Thus, 81% of all sites producing no-response errors during nTMS were concentrated on these areas.

In the action naming task, 34% of the sites associated with naming errors were located in the STG, 21% in the IFG, 12% in the PoG, 12% in the PrG, 12% in the SMG, 3% in the anG, and 3% in the MFG and in the MTG (Table 1 and Figure 2). A subanalysis by type showed that 53% of the sites associated with no-response errors (46% of all naming errors, see Figure 3C), were located in the STG, 20% in the PoG, and 13% in the IFG (Table 1). Thus, more than 80% of all sites producing no-response errors during nTMS were concentrated on these areas. Figure 2 depicts the sites where nTMS elicited naming errors in object and action naming tasks. Fused results for all subjects are shown in Figures 2A,B. Results for two individual subjects are shown in Figures 2C–F to reveal the inter-subject variability. Overall, the number, type, and location of the naming errors varied between the subjects.

The area-dependent subject-level analysis showed significant effects of nTMS in IFG, MFG, PoG, PrG, STG, MTG, and SMG for object naming (*p* < 0.05; see Table 2) and in IFG, PoG, STG, and SMG for action naming (*p* < 0.05; see Table 2). The most sensitive cortical sites were IFG, PoG, PrG, STG, and SMG (see Table 2 for summary). The largest difference of nTMS-sensitive sites in object and action naming tasks was in the PoG, where 7 subjects had a significant effect of nTMS on object naming and only one on action naming (Table 2). Clear individual differences between the subjects in the distributions of the speech-related areas were evident (Table 1 and Figure 2).

**Table 2:** **Single subject analysis within cortical areas**.

**Areas**	**Subjects (object/action)**								**Number of significant areas**
									**(sum over subjects)**
	**S1**	**S2**	**S3**	**S4**	**S5**	**S6**	**S7**	**S8**	
anG	-/-	-/-	-/-	-/-	-/-	-/-	-/-	-/-	0/0
IFG	0.005/0.004	0.002/-	0.001/-	-/-	-/0.001	0.005/-	-/-	0/-	5/2
MFG	0.005/-	-/-	-/-	-/-	-/-	-/-	-/-	-/-	1/0
PoG	0.005/-	0.001/-	-/-	0.046/-	0.001/-	0/-	0.001/0.019	0/-	7/1
PrG	0.001/-	0.002/-	0.001/-	-/-	-/-	0.001/-	-/-	0.037/-	5/0
STG	-/-	-/-	0/0	0/-	0.010/-	-/-	0/0	0.005/-	5/2
MTG	-/-	-/-	0.002/-	-/-	-/-	-/-	0/-	-/-	2/0
SMG	0.001/-	0.001/0.004	-/-	-/-	0.033/-	0.018/-	0.023/-	-/-	5/1

Areas where nTMS induced statistically significant effects on object and action naming. The p-values are indicated; “-” means p > 0.05 and “0” values < 0.001. Notation: anG, angular gyrus; IFG, inferior frontal gyrus; MFG, middle frontal gyrus; PoG, postcentral gyrus; PrG, precentral gyrus; STG, superior temporal gyrus; MTG, middle temporal gyrus; SMG, supramarginal gyrus.

In group-level analysis, the total number of nTMS-induced errors in object naming was significantly larger than in action naming (*p* = 0.002; see Table 1). No-response errors were significantly more frequent in object than action naming (*p* = 0.002); the number of semantic and phonological paraphasias did not differ significantly between the tasks. When the total number of errors within each gyrus was compared, object naming was more effectively disturbed by nTMS in PoG (*p* = 0.014) than action naming. No significant differences were observed for nTMS in the other gyri (Table 1).

## Discussion

We observed that object naming was consistently more disturbed by nTMS to the left hemisphere than action naming. The induced error types varied between subjects, but no-response errors were the most frequent in both tasks. In parallel with our results, object naming errors were more frequent than action naming errors during left-hemisphere DCS of neurological patients (Lubrano et al., 2014). Apparently, object naming is more sensitive to perturbations elicited by nTMS than action naming. DCS is probably more efficient than nTMS; in our study 3.2% of all trials induced naming errors, whereas 11.5% of the tested DCS sites were associated with induced language interferences (Lubrano et al., 2014). However, DCS mapping is limited by the extent of the craniotomy, and our nTMS speech mapping covered a wide cortical area. Hence, it is more likely to stimulate sites that are not speech-related in the nTMS than DCS mapping.

TMS induced naming errors from virtually all perisylvian sites (Figure 2). However, across subjects the location of these individual punctuate regions varied and there were no regionally specific effects of nTMS, which is in line with the results obtained by DCS studies (Corina et al., 2005; Lubrano et al., 2014). The classical Broca's area (Brodmann area 44/45) in IFG and the Wernicke's area (Brodmann area 22) in STG were both sensitive to nTMS in most subjects. It is evident, however, that the classical modular brain–language model is insufficient to explain our results. Instead, the results support the current state-of-the-art models of widely distributed language network (Poeppel and Hickok, 2004; Hagoort and Indefrey, 2014; Hope et al., 2014).

In our study, we did not measure the time-line aspect of language processing *per se*; instead we used repetitive TMS to induce speech disturbances. We assumed that our rTMS train was delivered early enough (from 300 ms onwards) to be able to disturb semantic processing, phonological code retrieval, syllabification, phonetic encoding, and articulation components of the language processing (Indefrey and Levelt, 2004; Indefrey, 2011; Strijkers and Costa, 2011) needed in overt object and action naming. However, as speech processing is not only sequential but probably also occurs in parallel during several phases of the processing, the specific identification of the affected processes seems unreliable.

Recently, 300 and 0 ms nTMS pulse train onsets were compared to study the effects on sensitivity and specificity of picture naming during language mapping with navigated TMS. The 0-ms onset produced more specific results in the parietal areas when compared to DCS data (Krieg et al., 2014). The 0-ms paradigm resembles more precisely the one applied in DCS so it is not surprising that the match between the 0-ms onset time and DCS is better. Nevertheless, the early onset of the nTMS may also influence conceptual preparation, lexical concept selection, and lemma retrieval attributed to the early stages of picture naming processes (Indefrey and Levelt, 2004; Indefrey, 2011). However, using the 300-ms latency for the nTMS pulse trains should have not biased our results to make object naming more sensitive to TMS than action naming, because action naming is a more demanding and time-taking process (e.g., Vigliocco et al., 2004; Mätzig et al., 2009).

Our results do not allow conclusions on cortical areas essential for processing of object-related or action-related words. Instead, they emphasize the network nature of language processing, in line with previous studies (Vigneau et al., 2006; Mätzig et al., 2009; Vigliocco et al., 2011; Lubrano et al., 2014). We did not confirm the previously described particular sensitivity of action naming for parietal cortical DCS (Corina et al., 2005). Our results were in line with more recent DCS results (Lubrano et al., 2014).

As the action naming was not specifically influenced by nTMS to posterior cortical areas, its use in preoperative speech mapping probably does not increase the sensitivity of nTMS in these regions. Therefore, the discordant results between nTMS and DCS of the posterior cortical areas (Picht et al., 2013; Tarapore et al., 2013), would probably not be improved by replacing the object naming with an action naming task.

Speakers name pictures of objects faster than those of actions, and action naming is more difficult than object naming in terms of accuracy and latencies (Vigliocco et al., 2002, 2004, 2011; Mätzig et al., 2009; Strijkers and Costa, 2011). This would suggest that action naming would be more easily disrupted by nTMS than object naming. However, the reverse was true in our experiment. Naming of words related with actions has been reported to involve more the motor cortex (Pulvermüller, 2005; Pulvermüller et al., 2005) and middle frontal gyrus (Lubrano et al., 2014). In our study, we did not stimulate those areas extensively enough to reach such conclusions. However, if the motor areas are more involved in action naming than object naming, it is possible that this “extra support” makes action naming less sensitive to TMS than object naming, when perisylvian regions are stimulated.

Object naming was particularly sensitive for nTMS to PoG, which is not typically studied by DCS when comparing object and action naming (Corina et al., 2005; Lubrano et al., 2014). However, in direct cortical recordings, spectral activity in PoG is modified during naming (Wu et al., 2011; Cogan et al., 2014). In fMRI, action naming induces stronger activation than object naming in PoG (Liljeström et al., 2008, 2009). It is possible that this stronger activation by action naming is less vulnerable to nTMS-induced perturbation.

Both fMRI and DCS have been used for language mapping. DCS during awake craniotomy is considered the gold standard for intraoperative brain mapping of cortical speech representations. However, it is demanding for the patient, strongly invasive, and may produce after-discharges, making the results difficult to interpret (Giussani et al., 2010). Moreover, the studied cortical regions are limited by the extent of craniotomy and demands of the surgery. Results from fMRI vary between different language paradigms and individuals, and its spatial accuracy in patients with gliomas has been questioned (Giussani et al., 2010; Wang et al., 2012). A recent case report suggests that nTMS may be more sensitive in defining speech lateralization than fMRI (Sollmann et al., 2013). The results of our study support the usefulness of picture naming combined with nTMS in presurgical planning (Krieg et al., 2013, 2014; Picht et al., 2013; Rösler et al., 2013). It also provides new possibilities for basic research of cortical speech representation. In addition, it may offer complementary information in comparison to other non-invasive methods (e.g., MEG and fMRI). Moreover, our results suggest that the efficacy of TMS in inducing naming errors can be modulated by the task; if a higher sensitivity is required, object naming is preferred; if a sparse amount of nTMS sites is required, action naming can be used.

Static pictures have limitations in exploring action naming performance, and some research groups have used videos of actions as stimuli to overcome this issue (e.g., Corina et al., 2005). Nevertheless, static images have been widely used in studies of action naming (see Mätzig et al., 2009). We did not match the frequency, familiarity length, or visual complexity of the pictures of objects vs. actions. Instead, subject-validated image stacks for objects and actions were obtained in the baseline naming session. It should be emphasized that we did not directly compare the naming of objects vs. actions, but we compared the sensitivity of fluently named objects or actions to nTMS (for a similar approach, see Lubrano et al., 2014).

In summary, we have compared the naming error distributions induced by nTMS during object and action naming tasks. We suggest that object naming is more easily disrupted by nTMS than action naming. Particularly nTMS to PoG induced more errors during object naming than during action naming. Thus, use of action naming instead of object naming tasks most likely would not improve the specificity of nTMS in mapping posterior speech-related areas (Picht et al., 2013; Tarapore et al., 2013). These features, however, can be used in varying the sensitivity of functional mapping by nTMS for different cognitive paradigms in basic research and for presurgical planning. To resume, TMS applied to 8 subjects induced 93 errors during object naming and 33 during action naming. We find this surprisingly convincing for relatively small material, but believe that increasing the number of subjects will provide further important information for cortical speech organization.

### Conflict of interest statement

The authors declare that the research was conducted in the absence of any commercial or financial relationships that could be construed as a potential conflict of interest.

## Supplementary material

Supplementary Video **Clips from baseline recordings and from errors in object and action 32 naming during navigated TMS recorded with experimental setup 1**.
