# Supplementary material for: Effects of navigated TMS on object and action naming
Source: Front Hum Neurosci. 2014 Sep 2;8:660. doi: 10.3389/fnhum.2014.00660 (PMC4151040; doi:10.3389/fnhum.2014.00660)
Supplement: Supplementary file 2 [file DataSheet1.DOC]

**Supplementary Table:** The number and percentage of pictures that were removed for object naming and action naming tasks after the baseline session.

| **Subjects** | **Removed pictures (object;action)** | |
| --- | --- | --- |
|  | No. | % |
| **S1** | 9; 12 | 9.2; 12.2 |
| **S2** | 4; 2 | 4.7; 2.0 |
| **S3** | 3; 5 | 3.1; 5.1 |
| **S4** | 13; 4 | 9.9; 4.1 |
| **S5** | 11; 4 | 8.4; 4.1 |
| **S6** | 25; 15 | 19.1; 15.3 |
| **S7** | 26; 23 | 19.8; 23.5 |
| **S8** | 25; 5 | 19.1; 5.1 |
